# Pharmacological Profiling of K_ATP_ Channel Modulators: An Outlook for New Treatment Opportunities for Migraine

**DOI:** 10.3390/ph16020225

**Published:** 2023-02-01

**Authors:** Tino Dyhring, Inger Jansen-Olesen, Palle Christophersen, Jes Olesen

**Affiliations:** 1 Saniona A/S, 2600 Glostrup, Denmark; 2 Danish Headache Center, Department of Neurology, University of Copenhagen, 2600 Glostrup, Denmark

**Keywords:** ATP-sensitive potassium channel, migraine, sulfonylurea receptor, thallium-flux, FLIPR

## Abstract

Migraine is a highly disabling pain disorder with huge socioeconomic and personal costs. It is genetically heterogenous leading to variability in response to current treatments and frequent lack of response. Thus, new treatment strategies are needed. A combination of preclinical and clinical data indicate that ATP-sensitive potassium (K_ATP_) channel inhibitors could be novel and highly effective drugs in the treatment of migraine. The subtype Kir6.1/SUR2B is of particular interest and inhibitors specific for this cranio-vascular K_ATP_ channel subtype may qualify as future migraine drugs. Historically, different technologies and methods have been undertaken to characterize K_ATP_ channel modulators and, therefore, a head-to-head comparison of potency and selectivity between the different K_ATP_ subtypes is difficult to assess. Here, we characterize available K_ATP_ channel activators and inhibitors in fluorescence-based thallium-flux assays using HEK293 cells stably expressing human Kir6.1/SUR2B, Kir6.2/SUR1, and Kir6.2/SUR2A K_ATP_ channels. Among the openers tested, levcromakalim, Y-26763, pinacidil, P-1075, ZM226600, ZD0947, and A-278637 showed preference for the K_ATP_ channel subtype Kir6.1/SUR2B, whereas BMS-191095, NN414, and VU0071306 demonstrated preferred activation of the Kir6.2/SUR1 subtype. In the group of K_ATP_ channel blockers, only Rosiglitazone and PNU-37783A showed selective inhibition of the Kir6.1/SUR2B subtype. PNU-37783A was stopped in clinical development and Rosiglitazone has a low potency for the vascular K_ATP_ channel subtype. Therefore, development of novel selective K_ATP_ channel blockers, having a benign side effect profile, are needed to clinically prove inhibition of Kir6.1/SUR2B as an effective migraine treatment.

## 1. Introduction

Migraine is a common neurological disorder in the general population. Migraine affects more than 1 billion people throughout the world, and it is the second most disabling of all diseases according to WHO [1,2]. It has huge socioeconomic and personal costs and, for these reasons, represents a vast need for drug treatment. The dominant feature of a migraine attack is headache. The exact mechanism of headache pain remains unknown but most likely involves the trigeminovascular system as the anatomical and physiological substrate from which nociceptive transmission originates and yields the perception of migraine. The existing treatments with triptans and non-selective prophylactic drugs are insufficient, and even with the recent advent of small molecule CGRP receptor antagonists and human monoclonal antibodies against CGRP or its receptor, despite representing a major advance, there is still a need for additional or better treatment options [3,4]. A target for such a treatment option could be the vascular ATP-sensitive potassium (K_ATP_) channel because it is expressed in migraine relevant tissues and dilates cephalic blood vessels [5]. Dilation of the dural vasculature, and especially the middle meningeal artery (MMA), has been implicated as one component underlying migraine. Accordingly, recordings of arterial circumference have demonstrated dilation of the MMA during migraine attack [6], and drugs shown to induce dilation of the MMA have been found to provoke headache in healthy volunteers [7,8,9]. More recently, it has been demonstrated that levcromakalim, a K_ATP_ channel activator, is the most effective agent experimentally inducing migraine attacks in migraineurs [10]. Furthermore, blocking K_ATP_ channels in rodent models of migraine is an effective pain-reducing treatment [11]. K_ATP_ channels are distributed in migraine related structures and more specifically Kir6.1 and SUR2B are expressed in cerebral and meningeal arteries and the trigeminal system [12,13]. Moreover, animal experimental studies indicate that the Kir6.1/SUR2B subtype is responsible for experimental induction of migraine-like changes [14]. Thus, the K_ATP_ channel subtype Kir6.1/SUR2B represents a promising target for the development of future migraine drugs.

K_ATP_ channels are large heteromeric protein complexes composed of four pore-forming inward rectifier K^+^ channel subunits (constituted by either Kir6.1 or Kir6.2) and four regulatory sulfonylurea receptor subunits (SUR1, SUR2A or SUR2B) [15]. K_ATP_ channels are expressed in most excitable tissues and are essential in numerous physiological processes including regulation of insulin secretion, control of vascular tone, and protection of cells against metabolic stress [16,17,18]. The channels are activated by Mg^2+^-bound nucleotides and ADP, which act on the SUR subunit, and inhibited upon binding of intracellular ATP to the Kir6 subunit. Thus, K_ATP_ channels are open during states of low metabolic activity, resulting in hyperpolarization of the plasma membrane. The ability to couple cellular metabolic state (ATP/ADP ratio) to electrical activity of the cell membrane is critical in numerous physiological processes and is a key feature of K_ATP_ channels [19]. Functional measurements, tissue mRNA and protein expression data, and analyses using transgenic animal models have identified Kir6.2/SUR1, Kir6.2/SUR2A, and Kir6.1/SUR2B as the major K_ATP_ channels and having a distinct tissue distribution in ß-cells, cardiac muscle, and vascular smooth muscle, respectively [20]. Furthermore, heterologous expression of Kir6 and SUR subunits in differing combinations reconstitutes different types of K_ATP_ channels with distinct electrophysiological properties and pharmacological sensitivities that reflect the various K_ATP_ channels in native tissues [21,22,23]. K_ATP_ channel activators and inhibitors have been in focus for pharmaceutical development for several decades for the treatment of diabetes, incontinence, arterial hypertension, asthma, and other disorders. Except for diabetes, these attempts have been clinically unsuccessful, but they have generated many compounds active on K_ATP_ channels. The aim of the present study was to analyze the efficacy of available activators and inhibitors at different human K_ATP_ channel subtypes using the same technology for alle substances. This is meant to be an initial step towards focusing on the development of specific inhibitors of the K_ATP_ channels subtype Kir6.1/SUR2B for the treatment of migraine.

## 2. Results

### 2.1. Functional Characterization of K_ATP_ Channel Activators

**Figure 1:**
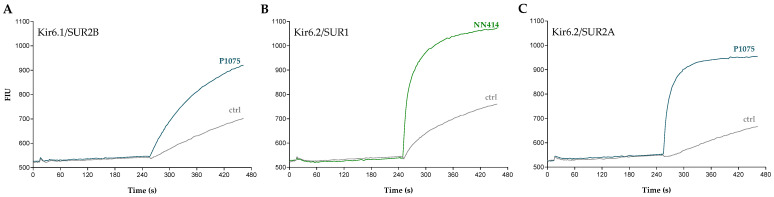
Recordings of K_ATP_ channel activation in thallium-flux assays. Representative fluorescence intensity traces demonstrating activation of (**A**) Kir6.1/SUR2B by 1 µM P1075, (**B**) Kir6.2/SUR1 by 3 µM NN414, and (**C**) Kir6.2/SUR2A by 10 µM P1075. The measured fluorescence intensity units (FIU) were recorded as a function of time and for each of the individual cell lines vehicle control traces are also depicted.

A series of K_ATP_ channel activators was tested at Kir6.1/SUR2B-, Kir6.2/SUR1-, and Kir6.2/SUR2A-HEK293 cells in fluorescence-based thallium-flux assays. A two-addition protocol was employed where various concentrations of individual test compounds were dispensed in the first addition and thallium buffer in the second addition. Figure 1 depicts FLIPR raw traces of cells expressing Kir6.1/SUR2B, Kir6.2/SUR1, or Kir6.2/SUR2A that were stimulated with either P1075 or NN414. Addition of these K_ATP_ channel openers resulted in an influx of Tl^+^ ions, which was seen as an increase in the fluorescence intensity. This increase was markedly larger than the background signal observed in the absence of the openers, demonstrating activation of the individual K_ATP_ channels.

**Figure 2:**
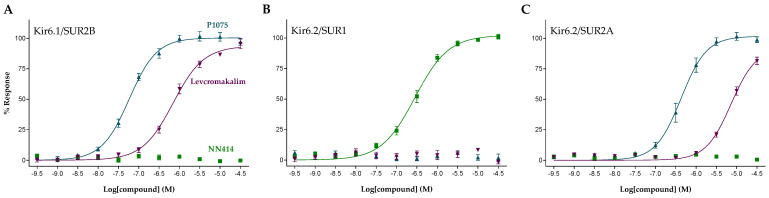
Concentration-response curves for P1075 (blue), levcromakalim (purple), and NN414 (green) at (**A**) Kir6.1/SUR2B, (**B**) Kir6.2/SUR1, and (**C**) Kir6.2/SUR2A. Symbols represent mean ± S.E.M.

The concentration–response relationship of the K_ATP_ channel activators P1075, levcromakalim, and NN414 at Kir6.1/SUR2B, Kir6.2/SUR1, and Kir6.2/SUR2A channels are shown in Figure 2. Whereas both P1075 and levcromakalim induced concentration-dependent activator responses at Kir6.1/SUR2B and Kir6.2/SUR2A, no effect was observed at Kir6.2/SUR1-HEK293 cells. This is in good alignment with previously reported findings on P1075 and levcromakalim being SUR2-specific K_ATP_ channel openers [24,25]. Likewise, NN414, that is reported to be a Kir6.2/SUR1-selective channel opener [26], demonstrated concentration-dependent activator responses when tested at the Kir6.2/SUR1-HEK293 cells but showed no activity at concentrations up to 30 µM when tested at Kir6.1/SUR2B and Kir6.2/SUR2A, thereby conforming its published selectivity profile. Calculated EC_50_ and maximal efficacy values of P1075, levcromakalim, and NN414, as well as additional reference K_ATP_ openers, are shown in Table 1. From these data it appears that five of the tested openers exhibit a potency selectivity that is greater than 1 log unit for Kir6.1/SUR2B over Kir6.2/SUR1 and Kir6.2/SUR2A. The rank order of selectivity for these compounds is ZD0947, ZM226600, Y-26763, pinacidil, and levcromakalim. Three openers had a marked selectivity for Kir6.2/SUR1 (NN414, VU0071063, and BMS-191095), whereas none of the tested compounds showed selectivity for Kir6.2/SUR2A.

### 2.2. Functional Characterization of K_ATP_ Channel Inhibitors

**Figure 3:**
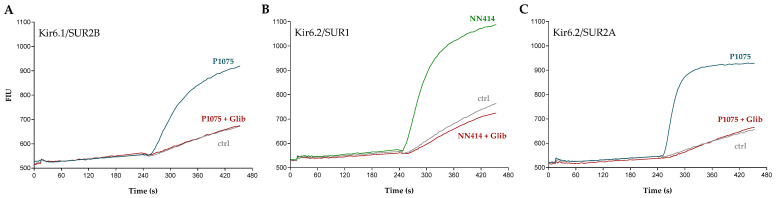
Recordings of K_ATP_ channel inhibition in thallium-flux assays. Representative fluorescence intensity traces in the absence or presence of glibenclamide (Glib, 1 µM) measured at (**A**) Kir6.1/SUR2B that was activated using 1 µM P1075, (**B**) Kir6.2/SUR1 that was activated using 3 µM NN414, and (**C**) Kir6.2/SUR2A that was activated using 10 µM P1075. The measured fluorescence intensity units (FIU) were recorded as a function of time and for each of the individual cell lines vehicle control traces are depicted as well.

A series of previously published K_ATP_ channel inhibitors were also characterized using a similar fluorescence-based thallium-flux methodology. Figure 3A shows FLIPR raw traces depicting Kir6.1/SUR2B-HEK293 cells that were stimulated with a nearly maximal effective concentration of 1 µM P1075. The response to 1 µM P1075 was comparable to the data presented in Figure 1A and demonstrates that activation by P1075 is immediate and does not require preincubation to exert its full effect. Cells preincubated with the K_ATP_ inhibitor glibenclamide (1 µM), prior to stimulation with P1075, demonstrated complete inhibition of Kir6.1/SUR2B channel activity. Similar responses were also obtained with Kir6.2/SUR1 and Kir6.2/SUR2A using 3 µM NN414 and 10 µM P1075 as activators, respectively (Figure 3B,C).

**Figure 4:**
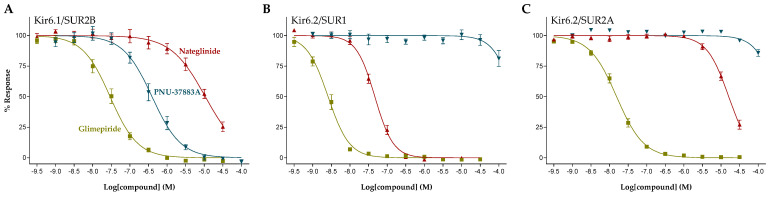
Concentration-response curves for glimepiride (yellow), PNU-37883A (blue), and nateglinide (red) at (**A**) Kir6.1/SUR2B, (**B**) Kir6.2/SUR1, and (**C**) Kir6.2/SUR2A. Symbols represent mean ± S.E.M.

The concentration-response relationship of the K_ATP_ channel inhibitors glimepiride, PNU-37883A, and nateglinide at Kir6.1/SUR2B, Kir6.2/SUR1, and Kir6.2/SUR2A channels are shown in Figure 4. The presented K_ATP_ channel inhibitors all display concentration-dependent inhibition although effective concentrations of the individual compounds vary between the three different K_ATP_ channel subtypes tested. Hence, glimepiride potently inhibited all three K_ATP_ channel subtypes with some preference for Kir6.2/SUR1, whereas nateglinide showed a more potent inhibition of the Kir6.2/SUR1 channel subtype, compared to the Kir6.1/SUR2B and Kir6.2/SUR2A K_ATP_ channel subtypes. In contrast, PNU-37883A demonstrated a clear selectivity profile towards Kir6.1/SUR2B. Calculated IC_50_ values are presented in Table 2. In addition to the three compounds presented in Figure 4, data on the sulfonylureas glibenclamide, gliquidone, gliclazide, and tolbutamide, repaglinide, and the thiazolidinediones troglitazone and rosiglitazone are presented as well. Both PNU-37883A and rosiglitazone exhibited a marked selectivity for Kir6.1/SUR2B and showed basically no inhibitory effect at concentrations up to 100 µM when tested at Kir6.2/SUR1 and Kir6,2/SUR2A. In contrast, nateglinide, gliquidone, and gliclazide demonstrated a selectivity profile with several orders of magnitude in favor of Kir6.2/SUR1, whereas none of the tested compounds showed a preferred selectivity for Kir6.2/SUR2A.

## 3. Discussion

By integrating cellular metabolism, membrane potential, and excitability, K_ATP_ channels carry out fundamental roles in nerve, muscle, epithelial, and endocrine tissue physiology [27]. Functional K_ATP_ channels have a tissue-specific subunit composition at the molecular level with distinct pharmacological and biophysical properties. This diversity of K_ATP_ channel properties, resulting from the differential molecular makeup, allows for exploitation of differential pharmacology which could lead to selective and tissue-targeted drugs.

Emerging evidence suggests the involvement of K_ATP_ channels in the pathophysiology of migraine. Although the cause and mechanisms underlying migraine are multifactorial and not yet fully understood, artery dilation in the dural vasculature is a key component mediated through membrane hyperpolarization caused by opening of potassium channels. K_ATP_ channels are expressed in migraine-related anatomical structures including vascular smooth muscle cells, trigeminal ganglion, and trigeminal nucleus caudalis [13,28,29], and recent research has demonstrated that activation of K_ATP_ channels triggers migraine attacks in migraineurs [10,30]. Drugs that inhibit K_ATP_ channels could, therefore, represent a novel migraine treatment principle. The K_ATP_ channel constituted by Kir6.1/SUR2B has, due to its dominant presence in vascular tissue, been suggested as a possible drug target for the treatment of migraine [11]. This is supported by recent data showing that mice with a conditional deletion of the K_ATP_ channel subunit Kir6.1 in smooth muscle cells exhibited a decreased arterial dilatory response to levcromakalim and did not develop the tactile hypersensitivity observed in wild-type mice following provocations with migraine-inducing drugs [14].

The present study was undertaken to conduct a detailed in vitro pharmacological characterization of a selection of available K_ATP_ channel activators and inhibitors. A series of previously published K_ATP_ channel openers were characterized in functional fluorescence-based thallium-flux assays using HEK293 cells stably expressing human Kir6.1/SUR2B, Kir6.2/SUR1, and Kir6.2/SUR2A K_ATP_ channels. These openers include benzopyrans (levcromakalim, Y-26763, and BMS-191095) [31,32,33], cyanoguanidines (pinacidil and P1075) [34,35], tertiary carbinols (ZM226600) [36], dihydropyridines (ZD0947 and A-278637) [37,38], benzothiadiazines (diazoxide and NN414) [26,39], and the xanthine derivative VU0071063 [40]. According to previously published data and the results presented in the present study, some selectivity between K_ATP_ channel subtypes has been introduced in the different chemical scaffolds. NN414, a potent analogue of diazoxide, is a K_ATP_ channel opener that demonstrates high selectivity for the Kir6.2/SUR1 subtype, which contrasts with the effects mediated by diazoxide. NN414 was shown to inhibit insulin secretion with a potency approximately 20 times higher than that seen with diazoxide treatment [41]. More recently, the xanthine derivate VU0071063 was identified in a fluorescence-based thallium-flux assay employing cells expressing Kir6.2/SUR1 [40]. VU0071063 activates native Kir6.2/SUR1 channels, thereby inhibiting glucose-stimulated calcium entry in isolated mouse pancreatic β cells. VU0071063 is reported to be selective for SUR1-containing K_ATP_ channels [40,42], a finding that was confirmed in the present study. Compounds with selectivity for Kir6.1/SUR2B include the chroman-substituted K_ATP_ openers where both levcromakalim and Y-26763 show preference for Kir6.1/SUR2B, and where BMS-191095 in contrast selectively activates Kir6.2/SUR1. Like levcromakalim, the cyanoguanidine derivatives pinacidil and P1075, the tertiary carbinol ZM226600 and the dihydropyridines ZD0947 and A-278637, demonstrate a selectivity preference for K_ATP_ channels constituting the SUR2A and SUR2B subunits. Levcromakalim, pinacidil, P1075, and ZM226600 have all demonstrated relaxation of basilar and middle cerebral arteries from rat via activation of K_ATP_ channels in vascular smooth muscle cells [28]. Interestingly, the potencies for the artery relaxation were equivalent to the potencies obtained at the expressed Kir6.1/SUR2B K_ATP_ channel subtype in the present study.

K_ATP_ inhibitors were also characterized in functional fluorescence-based thallium-flux assays using HEK293 cells expressing human Kir6.1/SUR2B, Kir6.2/SUR1, and Kir6.2/SUR2A. Glibenclamide, glimepiride, gliquidone, gliclazide, tolbutamide, repaglinide, and nateglinide all showed either equipotency or selectivity towards Kir6.2/SUR1. This finding is not surprising since the sulfonylureas and the meglitinides were developed as antidiabetic drugs and, hence, with preference for the pancreatic K_ATP_ channel subtype Kir6.2/SUR1. Glibenclamide was previously tested in two different rodent models of migraine. In the spontaneous trigeminal allodynic (STA) model, where a genetically inbred strain of rats exhibits tactile hypersensitivity in the head, glibenclamide increased the periorbital threshold to mechanical stimulation [11]. Similarly, in the mouse model of provoked migraine, glibenclamide was highly effective against glyceryl-trinitrate, cilostazol, and levcromakalim-induced tactile hypersensitivity [43], hereby adding to the conception of K_ATP_ channel inhibition as a novel mechanism to treat migraine. To test for a potential clinical effect of K_ATP_ channel inhibition in humans, two clinical trials were recently conducted in which the effect of systemic glibenclamide on cerebral blood flow and circumference of cranial arteries [44] and CGRP-induced headache and hemodynamic changes [45] were tested in healthy volunteers. In the study by Al-Karagholi and colleagues, it was reported that glibenclamide did not inhibit levcromakalim-induced vascular changes at a dose of 10 mg (p.o.). Lack of effect of glibenclamide was also reported by Coskun and colleagues as glibenclamide did not attenuate CGRP-induced headache and hemodynamic changes, again tested at a dose of 10 mg (p.o.). Unfortunately, higher doses have not been evaluated in humans probably due to a human dose limitation of glibenclamide, as higher doses could lead to severe hypoglycemia owing to glibenclamide’s high affinity to the Kir6.2/SUR1 subtype of K_ATP_ channels present in the pancreas. To evaluate the potential of K_ATP_ inhibitors for the treatment of migraine, it therefore seems important to develop new drugs selective for the Kir6.1/SUR2B subtype due to its dominant presence in vascular tissue.

In the present study, two compounds (rosiglitazone and PNU-37883A) were confirmed as being selective inhibitors of Kir6.1-containing K_ATP_ channels. The anti-diabetic rosiglitazone belongs to a class of thiazolidinediones (TZDs) that were developed to reduce insulin resistance in type II diabetes mellitus [46,47]. TZDs exert their properties by stimulating a nuclear hormone receptor, the peroxisome proliferator-activated receptor γ (PPARγ) [48]. Rosiglitazone has also been demonstrated to inhibit Kir6.1-containing K_ATP_ channels, although at somewhat higher test concentrations, by acting directly on the Kir6 subunit [49]. The drug was withdrawn from Europe (but not from the US) in 2010 following clinical reports indicating potential cardiovascular adverse effects and suggesting that rosiglitazone may increase the risk of heart failure. The association between TZDs and heart failure is well recognized as a class effect and an increased plasma volume rather than direct effects on cardiac function is thought to be the mechanism responsible for heart failure [50]. PNU-37883A is a morpholino-guanidine drug that was originally developed as a diuretic drug [51,52]. PNU-37883A was previously demonstrated to inhibit K_ATP_ channels in vascular smooth muscle at sub-micromolar concentrations with marginal effect at pancreatic, cardiac, or skeletal K_ATP_ channels even at high concentrations [52,53]. Like rosiglitazone, PNU-37883A mediates its inhibitory effects through the pore-forming Kir6 subunit [54]. However, higher systemic doses of PNU-37883A markedly depress cardiac function and as a result the development of the drug was stopped. Given the fact that K_ATP_ channels constituted by Kir6.1 and SUR2B are expressed in a wide range of vascular smooth muscle tissue throughout the body, it cannot be ruled out that some of the side effects reported on PNU-37883A is target mediated. However, although the specific mechanisms responsible for PNU-37883A’s effect on cardiac function remain speculative, the available evidence seems to discount a direct relationship with myocardial K_ATP_ blockade [55]. Hence, due to the present absence of clinically suitable substances, the discovery of novel potent and selective Kir6.1/SUR2B inhibitors is needed to test the therapeutic relevance of this target in human migraine.

## 4. Materials and Methods

### 4.1. Materials

BTC/AM was obtained from BioNordika (Herlev, Denmark). Diazoxide, levcromakalim, nateglinide, P1075, PNU-37883A, repaglinide, Y-26763, and ZM226600 were purchased from Tocris (Bristol, UK). BMS-191095, gliclazide, and glimepiride were purchased from MedChemExpress (Sollentuna, Sweden). All other chemicals were obtained from Sigma-Aldrich. Test compounds were dissolved in anhydrous DMSO to generate 10 mM stock solutions and diluted in assay buffer before use. The final concentration of DMSO used was less than or equal to 1 % (*v*/*v*).

### 4.2. Generation of Stable Cell Lines

Vectors containing DNA encoding human Kir6.1, Kir6.2, SUR1, SUR2A, and SUR2B subunits were purchased from GenScript Biotech (Rijswijk, The Netherlands). HEK293 cells (ATCC, Manassas, VA, USA) were cultured in DMEM (Life Technologies, Roskilde, Denmark) supplemented with 10% fetal calf serum (Invitrogen, Allerød, Denmark). HEK293 cells were transfected with Kir6.x and SURx expression plasmids using lipofectamine (Invitrogen, Denmark) according to manufacturer’s instructions. Transfected cells were selected in medium supplemented with 250 µg/mL zeocin and 150 µg/mL hygromycin (Life Technologies, Denmark). Single clones were picked and propagated in selection media until sufficient cells were available for freezing. Expression of functional K_ATP_ channels were verified in fluorescence-based thallium-flux assays.

### 4.3. Thallium-Flux Assays

Thallium-flux assays were performed essentially as described previously [56]. Briefly, stably transfected HEK293 cells expressing either Kir6.1/SUR2B, Kir6.2/SUR1, or Kir6.2/SUR2A were cultured overnight in poly-D-lysine-coated 384-well optiplates (Corning, Amsterdam, The Netherlands) at a density of approximately 30,000 cells/well. On the day of the experiment, the cell culture medium was aspirated, and the cells washed once with Cl^-^ free assay buffer (in mM: 140 sodium gluconate, 7.5 potassium gluconate, 6 calcium gluconate 1 magnesium gluconate, 5 glucose, 10 HEPES, pH 7.3) using an Aquamax 4000 (Molecular Devices, Sunnyvale, CA, USA). After washing, the Cl^−^ free assay buffer was replaced with 25 µL/well of 2 µM of the thallium-sensitive dye BTC/AM in Cl^-^ free assay buffer supplemented with 2 mM amaranth and 1 mM tartrazine. After 1-h incubation at 37 °C in a humidified 5% CO_2_ environment, the plates were transferred to a FLIPR Penta (Molecular Devices, Sunnyvale, CA, USA). Both K_ATP_ channel activators and inhibitors were tested in a two-addition protocol. Following an initial baseline recording to assess the background fluorescence, test compounds were dispensed at different concentrations to individual wells of the assay plate. After an additional 4 min of recording, a stimulus buffer (Cl^-^ free assay buffer supplemented with 2 mM amaranth, 1 mM tartrazine, and 1 mM Tl_2_SO_4_) was added to the wells. When testing K_ATP_ inhibitors, the stimulus buffer also contained a K_ATP_ opener at a nearly maximal effective concentration (Kir6.1/SUR2B, 1 µM P1075; Kir6.2/SUR1, 3 µM NN414; Kir6.2/SUR2A, 10 µM P1075).

### 4.4. Data and Statistical Analysis

Responses were measured as area under the curve (AUC) that was background corrected for basal influx of thallium. Curve fitting and parameter estimation were carried out using GraphPad Prism 9 (GraphPad Software Inc., San Diego, CA, USA). Data are expressed as mean ± S.E.M.

## 5. Conclusions

The present study has characterized existing K_ATP_ channel modulators using the same technology for all compounds. Both K_ATP_ channel activators and inhibitors, with diverse chemical and structural properties, display varying degrees of channel subtype-selectivity which can be utilized for tissue-specific targeting with both research and therapeutic perspectives. However, our results also highlight that there are currently no selective Kir6.1/SUR2B inhibitors available for studies of human migraine. While a convincing amount of preclinical evidence suggests that the K_ATP_ channel subtype Kir6.1/SUR2B is a promising drug target for migraine, translation to patients is pending better pharmacological tools and novel selective inhibitors with a benign side effect profile are needed to prove or disprove this interesting drug target.

## Figures and Tables

**Table 1:** Selectivity profiling of reference K_ATP_ activators.

Compound	Structure	Kir6.1/SUR2B		Kir6.2/SUR1		Kir6.2/SUR2A	
		EC_50_ (µM)	Efficacy (%)	EC_50_ (µM)	Efficacy (%)	EC_50_ (µM)	Efficacy (%)
Levcromakalim	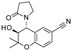	0.55 ± 0.29 (7)	90 ± 6 (7)	>30 (7)	0 (7)	6.9 ± 2.2 (7)	85 ± 11 (7)
Y-26763	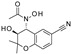	0.075 ± 0.042 (6)	96 ± 3 (6)	4.1 ± 2.1 (7)	98 ± 2 (7)	1.3 ± 0.97 (7)	71 ± 5 (7)
BMS-191095	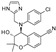	>30 (5)	0 (5)	1.4 ± 0.95 (5)	82 ± 10 (5)	>30 (6)	0 (6)
Pinacidil	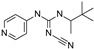	1.1 ± 0.60 (6)	76 ± 9 (6)	>30 (6)	0 (6)	17 ± 5.8 (6)	73 ± 14 (6)
P1075	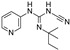	0.052 ± 0.024 (10)	100 (10)	>30 (10)	0 (10)	0.51 ± 0.27 (10)	100 (10)
ZM226600	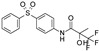	0.15 ± 0.090 (5)	87 ± 6 (5)	>30 (5)	0 (5)	2.8 ± 2.2 (5)	54 ± 10 (5)
ZD0947	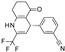	0.52 ± 0.22 (6)	57 ± 2 (6)	>30 (6)	0 (6)	11 ± 5.9 (6)	33 ± 9 (6)
A-278637	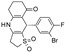	0.17 ± 0.089 (5)	88 ± 6 (5)	>30 (6)	0 (6)	1.6 ± 0.89 (6)	95 ± 4 (6)
NN414	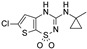	>30 (9)	0 (9)	0.26 ± 0.16 (9)	100 (9)	>30 (9)	0 (9)
Diazoxide	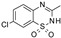	9.7 ± 5.7 (7)	38 ± 9 (7)	6.2 ± 4.8 (7)	97 ± 5 (7)	>100 (7)	0 (7)
VU0071063	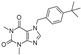	>30 (5)	0 (5)	1.1 ± 0.62 (6)	100 ± 1 (6)	>30 (6)	0 (6)

Functional properties of K_ATP_ channel activators were characterized at human Kir6.1/SUR2B, Kir6.2/SUR1, and Kir6.2/SUR2A channels stably expressed in HEK293 cells. Fitted efficacy levels were normalized to the maximal efficacy obtained with P1075 (Kir6.1/SUR2B and Kir6.2/SUR2A) and NN414 (Kir6.2/SUR1). The values are given as mean ± S.D. and the number of individual experiments (each performed in quadruplicate) are in parentheses (n).

**Table 2:** Selectivity profiling of reference K_ATP_ inhibitors.

Compound	Structure	Kir6.1/SUR2B	Kir6.2/SUR1	Kir6.2/SUR2A
		IC_50_ (µM)	IC_50_ (µM)	IC_50_ (µM)
Glibenclamide	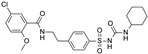	0.021 ± 0.014 (5)	0.00087 ± 0.0005 (5)	0.0099 ± 0.0041 (5)
Glimepiride	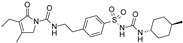	0.033 ± 0.023 (6)	0.0023 ± 0.0012 (6)	0.017 ± 0.009 (5)
Gliquidone	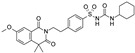	4.5 ± 3.6 (6)	0.0069 ± 0.0050 (6)	0.78 ± 0.58 (6)
Gliclazide	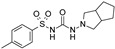	24 ± 11 (5)	0.61 ± 0.45 (7)	55 ± 22 (6)
Tolbutamide	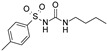	41 ± 11 (5)	7.5 ± 4.1 (6)	71 ± 12 (5)
Repaglinide	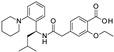	0.0011 ± 0.0006 (5)	0.0039 ± 0.0014 (5)	0.00094 ± 0.00046 (5)
Nateglinide	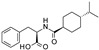	14 ± 6.7 (6)	0.044 ± 0.013 (5)	16 ± 7.3 (5)
Troglitazone	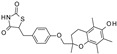	10 ± 6.5 (8)	14 ± 3.1 (5)	30 ± 16 (5)
Rosiglitazone	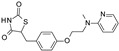	8.5 ± 4.6 (6)	>100 (5)	>100 (5)
PNU-37883A	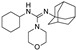	0.29 ± 0.21 (6)	>100 (6)	>100 (6)

Functional properties of K_ATP_ channel inhibitors were characterized at human Kir6.1/SUR2B, Kir6.2/SUR1, and Kir6.2/SUR2A channels stably expressed in HEK293 cells. The values are given as mean ± S.D. and the number of individual experiments (each performed in quadruplicate) are in parentheses (n).

## Data Availability

Data is contained within the article.
